# A rapid review of the evidence for online interventions for bereavement support

**DOI:** 10.1177/02692163241285101

**Published:** 2024-10-15

**Authors:** Anne Finucane, Anne Canny, Ally Pax Arcari Mair, Emily Harrop, Lucy E Selman, Brooke Swash, Donna Wakefield, David Gillanders

**Affiliations:** 1 Clinical Psychology, School of Health in Social Science, University of Edinburgh, Edinburgh, UK; 2 Marie Curie Hospice Edinburgh, Edinburgh, UK; 3 Marie Curie Research Centre, Division of Population Medicine, School of Medicine, Cardiff University, Cardiff, UK; 4 Palliative and End of Life Care Research Group, Population Health Sciences, Bristol Medical School, University of Bristol, Bristol, UK; 5 University of Chester, Chester, UK; 6 North Tees and Hartlepool NHS Foundation Trust, Stockton, UK; 7 Population Health Sciences Institute, Faculty of Medical Sciences, Newcastle University, Newcastle, UK

**Keywords:** Grief, bereavement, adjustment disorders, social support, psychological support, psychosocial support system, internet-based intervention, telemedicine, digital health

## Abstract

**Background:**

Grieving is a natural process, and many people adjust with support from family and friends. Around 40% of people would benefit from additional input. Online bereavement support interventions may increase access to support. Evidence regarding their acceptability and effectiveness is emerging but needs to be synthesised.

**Aim:**

To synthesise evidence on the feasibility, acceptability, effectiveness, impacts and implementation of online interventions to improve wellbeing, coping and quality of life after bereavement.

**Design:**

A rapid review of evidence regarding online bereavement support. We appraised study quality using AMSTAR 2 and the Mixed Methods Appraisal Tool.

**Data sources:**

English language articles published 1 January 2010 to 4 January 2024, using Ovid MEDLINE, Ovid Embase and APA PsycINFO. Eligible articles examined formal and informal online interventions to improve bereavement outcomes.

**Results:**

We screened 2050 articles by title and abstract. Four systematic reviews and 35 individual studies were included. Online bereavement support was feasible, acceptable and effective in reducing grief intensity, stress-related outcomes and depression. Where reported, participant retention was typically >70%. Positive impacts included: access to a supportive community at any time, reduced isolation; opportunities to process feelings; normalisation of loss responses; access to coping advice and opportunities for meaning-making and remembrance. Negative impacts included upset due to insensitive comments from others via unmoderated online forums.

**Conclusion:**

Online interventions can widen access to acceptable, effective bereavement support and improve outcomes for bereaved people. National policies and clinical guidelines relating to bereavement support need to be updated to take account of online formats.

**What is already known about this topic?**

- Approximately 60% of people who are bereaved adjust with only the support of family and friends, whilst around 40% require additional support with their grief.
- Online and digital interventions to support bereaved people are rapidly developing and show promise as a way to meet increased demand.

**What this paper adds?**

- Online bereavement support is feasible, acceptable and effective in improving grief intensity, reducing stress-related outcomes and depression.
- Online bereavement support can enable access to a supportive community, reduce isolation, provide opportunities to express and process emotions, normalise grief responses, give access to information on adaptive coping and provide opportunities for adaptive meaning-making.
- Negative aspects of online bereavement support include technical issues accessing support, insensitive comments via unmoderated online forums or distress triggered by reading other people’s experiences of loss.

**Implications for practice, theory and policy**

- Online bereavement support interventions including self-directed, volunteer-led, and formal psychological interventions, delivered online by mental health specialists, have much potential in ensuring that differing levels of bereavement support needs experienced by individuals can be met.
- Challenges to accessing online support and potential negative impacts should be taken into consideration when developing a new online bereavement support intervention.
- Future research should investigate the value of distinct forms of online bereavement support for more diverse populations, assess its cost-effectiveness, and determine optimal levels of facilitation for different levels of need.

## Background

Grieving is a natural process, and typically 60% of people who are bereaved adjust with support from family and friends. Approximately 40% need additional support, including 6%–10% who experience symptoms of pathological grief.^1,2^ Since the COVID-19 pandemic, demand for bereavement support has increased, as has the complexity of need.^3^ The increase in the prevalence of grief symptoms and disorders since the COVID-19 pandemic is a global phenomenon, with a meta-analysis of 15 studies involving 9289 participants across several countries estimating a pooled prevalence rate of grief disorder at 46%.^4^ A UK-based longitudinal study of 711 bereaved individuals found that the proportion of people meeting the threshold for posttraumatic grief disorder was higher following the COVID-19 pandemic compared with pre-pandemic times, with over one-third of people bereaved showing these symptoms at 13 months following bereavement.^5^ Loneliness, social isolation, disruption to typical mourning practices and limited access to support is associated with this increase,^3–6^ and has contributed to the growing need for accessible evidence-based bereavement support.

Healthcare organisations and practitioners increasingly recognise the importance of digital health interventions in improving access to quality healthcare services and support.^7^ Smartphones, video-conferencing, websites, apps and other virtual and online resources are particularly valuable for people in remote and rural locations, and those who need access to support at any hour.^8–10^ In palliative and end-of-life care contexts, such interventions are thought to widen access to support in a cost-effective manner, with evidence indicating positive impacts on education, information sharing, decision-making and communication.^11^

Digital interventions used to improve wellbeing and quality of life after bereavement are rapidly being developed and evaluated. Emerging evidence from four systematic reviews indicates acceptability and perceived value of online support for bereaved populations.^12–15^ However, these previous reviews were focussed on specific populations such as those bereaved by suicide;^14^ specific forms of support such as online forums or support groups^13,14^ or Cognitive Behavioural Therapy.^12,15^ So, while they provide valuable detail in relation to specific populations and interventions, they are limited in scope. Critically, all four previous reviews were published before, or at the start of the COVID-19 pandemic, thus only include data collected before the COVID-19 pandemic. Due to radical changes in the design of health and bereavement services during the pandemic, with the rapid introduction of telemedicine, virtual consultations and online support, the way in which people accessed support changed. People became more used to accessing healthcare and bereavement support remotely.^16–18^ Given this seismic shift in how health and bereavement care is accessed, a review of the evidence pertaining to the value of online bereavement support interventions that takes account of a wider range of populations, and also includes studies conducted during and since the COVID-19 pandemic, is needed to guide future intervention development.

We aimed to synthesise published evidence on the development, feasibility, effectiveness, impacts and implementation of online interventions for people who have been bereaved. Our aims were broad as we were interested in any evidence that would support the development of online bereavement support, including our own novel online bereavement support intervention (My Grief My Way).^19^ To ensure our findings would be of interest to service commissioners, we included evidence on interventions designed to meet the needs of people with bereavement support needs that could not be met by their own family, friends and local networks. We were also keen to examine evidence on implementation, given the recognition of the need to consider factors that influence implementation from the early stages of intervention development.^20^ We viewed the earlier four reviews as a starting point, but sought to draw on a wider range of populations, interventions and study designs to meet our aims. Our research questions were:

i. What types and formats of online interventions for bereavement support have been evaluated, and what is the evidence for their effectiveness and impacts?
ii. For which populations have online bereavement support interventions been evaluated and what evidence exists on their effectiveness?
iii. What is the evidence on the feasibility and acceptability of online interventions for bereavement?
iv. What are the barriers and facilitators regarding implementing online bereavement support?
v. What type of outcomes have been assessed in research examining online interventions for bereavement support, and what comparators have been used?

## Methods

### Design

We undertook a rapid integrative review of the evidence for online interventions for bereavement support. Rapid reviews are an efficient approach to evidence synthesis, ensuring that findings can be quickly made available to decision-makers.^21^ We chose a rapid review design so that evidence could quickly be generated and synthesised to inform the development of our My Grief My Way online bereavement support intervention (ISRCTN: 18357870).^19^ We conducted a rapid integrative review as we were interested in evidence generated from diverse methodologies.^22^

To ensure rigour, we followed interim guidance for rapid reviews.^21,23^ In line with this, we established an experienced team in conducting systematic reviews; we registered our protocol; we searched at least two electronic databases; we involved more than one reviewer in article screening; we ensured all data extracted were cross-checked by a second reviewer; we ensured each article was independently appraised for quality by two reviewers; we used Covidence (software) to streamline the process; and we provided a comprehensive knowledge synthesis describing results, quality of evidence and implications.26 We sought feedback on findings from our My Grief My Way^19^ stakeholder group, which included organisations involved in designing and delivering bereavement support, as well as public representatives with experience of grief.

### Search strategy

We use the PICO (Population, Intervention, Context, Outcome) mnemonic to refine the search strategy, research questions and the inclusion and exclusion criteria (Table 1).^24^ We searched three databases: Ovid MEDLINE, Ovid Embase and APA PsychInfo to identify papers published between 1st January 2010 and 11th January 2023. We decided to include articles published prior to the pandemic, as our research questions were broader than those covered in existing reviews, and this allowed for a more comprehensive synthesis of evidence. We used 2010 as a starting point as smartphones started to become popular from this period onwards, and there was a consensus within the research team that evidence prior to this may have less relevance given the rapid technological changes since. We updated the search in January 2024 to add studies published between 11th January 2023 to 4th January 2024. All searches were undertaken by AF. We included primary research and review papers in the English language only, due to time constraints and a lack of translation resources. Commentaries, protocols and editorials were excluded. We also searched PROSPERO and ISRCTN for information on current studies and reviews in progress. The review protocol was registered with PROSPERO on 3rd March 2023.^25^

**Table 1.** PICO components and inclusion/exclusion criteria.

Population	Bereaved carers, family members, children, chosen-family, friends and colleagues including those bereaved due to short or long-term illness, sudden or accidental death, peri-natal death (including miscarriage) and suicide. Professional staff (e.g. care home or palliative care staff) who have participated in a grief support intervention.	
Intervention	Online interventions were web-based or internet interventions delivered via personal computer, tablet or smartphone with the aim of improving outcomes for people who have been bereaved.	
Comparison	Any comparison including treatment as usual; waiting list control, pre versus post intervention. For qualitative studies, a comparison was not required – qualitative studies focussing on the feasibility, effectiveness or implementation of an online bereavement support intervention were included. For quantitative studies describing relationships between key variables, a comparison was not required.	
Outcome	Any psychological or psychosocial outcome including well-being, coping, distress, depression, quality of life, posttraumatic stress, posttraumatic growth, meaning and purpose.	
Inclusion criteria		Exclusion criteria
I	Studies focussed on bereaved carers, family members, chosen-family, friends or colleagues. This could include those bereaved due to short or long-term illness, sudden death (including accident), perinatal death (including miscarriage) and suicide.	Interventions that are *exclusively* telephone-based with no online component. Studies of digital memorialisation. SMS text-only interventions.
ii.	Studies focussed on an online intervention for bereavement support, or a mix of telephone and online support.	Web-based surveys describing general grief experiences that are not focussed on a specific intervention.
iii.	Qualitative, quantitative, mix-method or systematic review studies evaluating an online bereavement support intervention or describing its development or implementation. Eligible systematic review studies included systematic, scoping, rapid and integrative reviews.	Narrative reviews, book chapters, comment papers, case reports, conference abstracts and PhD dissertations. Case studies, or studies with papers describing interventions with no evaluation.
iv.	Studies describing online interventions (in the broad sense), where research data has been collected to describe feasibility, impact, acceptability, effectiveness or implementation.	Study protocols, general descriptions of interventions with no evaluation.
v.	Studies describing interventions focussed on improving psychological/psychosocial outcomes for bereaved populations or describing the development, feasibility or implementation of such interventions.	Interventions targeting only anticipatory grief before a bereavement.
vi.	English language.	Languages other than English.
viii.	Online interventions included formal psychological, psychosocial and psychoeducational interventions delivered by professionals, as well as informal peer support interventions such as online communities, Facebook groups and online bereavement support resources Interventions included those involving bereavement services, mental health services, psychological support services or specialist counselling/psychotherapy services. We included online bereavement programmes, online volunteer-led bereavement support, online self-help groups, online community groups (e.g. Facebook groups) or online bereavement support resources. We included both individual and group interventions, as well as self-directed interventions and those with a mix of delivery modes.	Interventions such as online practical and emotional support provided by family, friends, work colleagues and other members of the bereaved person’s social network.

### Study selection

We included studies evaluating online bereavement support for anyone who had been bereaved, irrespective of their relationship to the person who died, the type of bereavement or when this occurred (Table 1). We included all types of research designs and systematic reviews. While systematic reviews are often excluded from updated reviews, Cochrane rapid review recommendations suggest including systematic reviews as a relevant study design.^21^ We also included informal interventions, such as online forums, if these were formally evaluated.

### Data extraction

Search results were uploaded to Covidence, an online platform that supports systematic data handling for systematic review studies.^26^ Duplicates were automatically removed by the Covidence software. A team of six researchers independently screened article titles and abstracts (AC, AF, APAM, DW, BS and EH) before progressing to independent full text review by two researchers (AC, AF, EH, DW, BS and DG). All abstracts were independently screened at least twice. Where areas of conflict were identified, consensus was reached through consulting a third researcher or the wider team. Data were extracted from individual studies by one member of the review team and cross-checked by another. Data extracted were saved in an MS Excel file, which contained one worksheet for systematic review studies and another for individual studies. Data extraction headings are outlined in Table 2. Data from individual studies previously reported in systematic reviews were not extracted,^21^ instead we extracted data from the systematic review in its entirety.

**Table 2.** Data extracted from included studies.

Data extracted from review papers	Data extracted from papers describing individual studies that were not included in a previous review
• Author(s). • Publication Year. • Title. • Country (where data collection for the review took place). • Aim. • Population (i.e. target of the intervention). • Whether a risk of bias assessment was undertaken, and instrument used. • Number of studies included and dates. • Types of studies included. • Key findings. • Review author conclusions.	• Author(s). • Publication Year. • Title. • Country (where data collection took place). • Aim. • Study design. • Population (i.e. target of the intervention). • Mode of intervention delivery. • Description of intervention. • Formal or informal intervention. • Intervention format. • Participant recruitment rates and retention. • Outcomes assessed (including time points) and comparators where applicable. • Results. • Reported limitations and strengths. • Implications for future online bereavement support studies. • Source of funding.

### Quality appraisal

We used the AMSTAR (A MeaSurement Tool to Assess Systematic Reviews) Version 2^27^ to assess the quality of systematic reviews, and the MMAT (Mixed Methods Appraisal Tool)^28^ to appraise all other studies. Each article was assessed independently by two members of the research team (AC, AF, APAM, DW, EH or BS). Disagreements were discussed to reach consensus, involving a third reviewer if needed.

### Data analysis and synthesis

The first author (AF) conducted a narrative synthesis organised around our research questions. This was descriptive in nature in line with recommendations for rapid reviews.^21,23^ MS Excel pivot tables were used to tabulate key characteristics of each study, alongside a summary of the quantitative and/or qualitative findings. Systematic review studies were tabulated and described separately from individual studies due to the different design and approach to quality appraisal. All papers were downloaded into NVivo 12 alongside the data extraction tables. Our analysis was informed by a framework approach,^29^ whereby we sought to deductively code study findings in relation to pre-existing categories relevant to our research questions: intervention feasibility and acceptability, effectiveness, impacts and factors relating to implementation to guide future online intervention development. NVivo search and explore functions were used to support this process. A meta-analysis was not performed given the heterogeneity of study designs, interventions and outcomes.

## Results

### Overview

**Figure 1.**
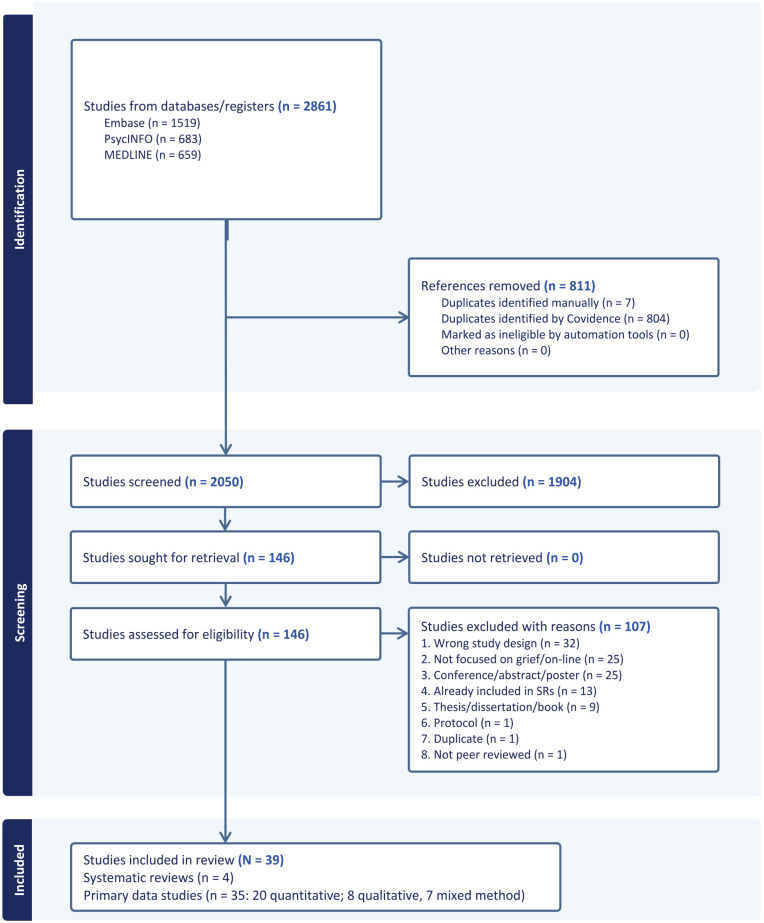
PRISMA flow chart.^30^

A total of 2050 article titles and abstracts were screened (Figure 1). From these, 1904 papers were excluded, leaving 146 papers for full text review. Following a full text review of all papers, a further 107 papers were excluded as they did not meet study criteria (*n* = 94) or had been previously reported in the included systematic reviews (*n* = 13). Four systematic reviews (Table 3) and 35 primary studies (Table 4) were included in the synthesis.

**Table 3.** Overview of systematic review studies (*n* = 4).

Author/country	Aim	Population	Number and design of studies	Type of interventions	Findings
Lestienne et al.^14^ France/Australia	To examine the use and benefits of online resources dedicated to people bereaved by suicide and appraise the quality of the research in this field.	People bereaved by suicide.	12 studies published between 2008 and 2020: Qualitative (*n* = 4) Quantitative (*n* = 4) Mixed method (*n* = 4)	Online support groups (*n* = 8) Online memorials (*n* = 5) Facebook (*n* = 3) Online booklet (*n* = 1) *Some studies included more than one intervention type.	*• AMSTAR rating: moderate quality.* • People bereaved by suicide use online resources to seek and share support and information. • Online spaces are also used to memorialise loved ones and for meaning-making. • Online resources were predominantly used by middle-aged women, parents who lost their child by suicide and recently bereaved individuals. • Online resources support help-seeking at any hour for socially disadvantaged, and isolated people. • May enhance early access to help and support – value in having access to a resource shortly after the loss. • Negative effects were rare. • Online resources are an essential addition to available, in person resources.
Robinson et al.^13^ New Zealand	To determine whether online support groups benefit people who have been bereaved.	People who have been bereaved.	9 studies published between 2001 and 2016 Qualitative (*n* = 5) Quantitative (*n* = 2) Mixed method (*n* = 2)	Online support groups including chatrooms, email groups and Facebook groups.	*• AMSTAR rating: moderate quality.* • People who use online support groups value their experiences in these communities. • Users valued being able to access support at any time and being able to share their experiences with others who have had similar experiences. • Online support groups are especially helpful when there is stigma attached to the loss. • Online support groups provided a sense of community, emotional support, sharing of information, reconstruction of a sense of identity, involvement in a remembrance process and a realisation of the changing nature of grief over time. • Few negative experiences were reported, but mainly related to miscommunications, technical issues, distress from reading others’ messages, though benefits appeared to outweigh these. • Insufficient high quality quantitative evidence to determine whether online support groups reduce grief symptoms and improve wellbeing.
Wagner et al.^15^ Germany	To review the evidence for web-based interventions for bereaved people.	Adults who have experienced any types of loss of a significant person.	7 studies published between 2006 and 2015 RCT (*n* = 7) using active or waitlist control groups.	Intervention based on cognitive behavioural therapy (CBT). Included a mix of unguided and part-guided structured writing assignments.	*• AMSTAR rating: low quality.* • Online CBT showed moderate to large effects on symptoms of grief and posttraumatic stress disorder. • Impact on depression was statistically significant, but the effect size was small. • Average attrition rate was 27%. Some participants dropped out as the intervention was too impersonal or lacked personal feedback. • 4 of the 7 studies assessed as ‘good quality’.
Zuelke et al.^12^ Germany	To synthesise evidence on the effectiveness and feasibility of internet and mobile-based interventions for symptoms of grief after bereavement.	Adults (⩾18 years) who experienced bereavement.	9 studies published between 2004 and 2019. RCT (*n* = 7) Follow-up (*n* = 1) Pilot (*n* = 1)	Online intervention based on CBT or social cognitive theory. Included a mix of unguided, email based and telephone-based studies.	*• AMSTAR rating: high quality.* • Internet or mobile-based interventions for grief after bereavement were found to be effective against symptoms of grief, posttraumatic stress syndrome (PTSS) and depression, with the largest effect sizes observed for PTSS. • Effects observed were smaller for depression than for grief symptoms. • User satisfaction was rated as moderate to high.

**Table 4.** Overview of original studies (*n* = 35).

Author/country	Aim	Study design	Participants	Intervention type	Findings
Brodbeck et al.^31^ Switzerland	To examine Emotional Regulation (ER) and loss-related Coping Self-Efficacy (CSE) using internet intervention for prolonged grief symptoms	Quantitative Mediation analysis	Older adults who had experienced loss due to bereavement or separation or divorce (*n* = 20 of 100 had experienced spousal bereavement).	Psychological (CBT). Comprised 10 text-based self-directed modules and a weekly email as guidance.	• CBT intervention increased both ER and CSE, which correlated with improvements in grief and psychopathology symptoms (β = 0.33; *p* = 0.001) and loss-related coping self-efficacy (β = 0.30; *p* = 0.002), both of which correlated with improvements in grief and psychopathology symptoms.
Chang et al.^32^ USA	To address challenges that are unique to delivering group-counselling services over videoconference technology in a rural area.	Mixed methods	Bereaved adults living in Rural Texas (*n* = 10).	Psychosocial/psychoeducational. Delivered by psychologists or mental health specialists, based on 5 stages of grief.	• Clinicians felt there was value and benefit for the group members, regardless of whether or not the members met criteria for complicated grief. • Online group counselling for grief can be conducted successfully for people in rural areas, areas and those on low-income. • Support was perceived as good as face-to-face service and that the group helped them deal more effectively with their problems.
Cipolletta et al.^33^ Italy	To explore how live chat can support survivors in their bereavement process.	Qualitative	People bereaved by suicide (*n* = 30).	Psychosocial. Delivered by volunteers, supervised by a psychologist.	• Five themes identified: meaning-making, reactions to the loss, resources, needs and interactions with the operator. • Survivors used the live chat as a safe space in which to disclose non-socially desirable details and to make sense of suicide through the reconstruction of events and the deceased’s’ motivations.
Dias et al.^34^ USA	To develop a web-based intervention for bereaved parents to meet their individualised needs in order to improve health outcomes.	Qualitative	Comprised 3 groups (*n* = 14): Bereaved parents of a child 0–18 years (*n* = 2), paediatric healthcare providers (*n* = 8), IT specialists, (*n* = 4).	Psychological/psychoeducational/psychosocial. Delivered by counsellors or mental health specialists.	• Intervention generally acceptable. • Data highlighted concerns that may influence scalability: internet access, access to the specific resources, diversity in the population. • Both literacy level and language were noted to be potential problems.
Dominguez-Rodriguez et al.^35^ Ecuador/Spain/Netherlands/Mexico	To evaluate the efficacy of a web-based treatment, Grief COVID (Duelo COVID in Spanish), in reducing clinical symptoms of complicated grief, depression, posttraumatic stress, hopelessness, anxiety and suicidal risk in adults.	Quantitative RCT (intervention v waitlist control)	Adults bereaved during COVID (*n* = 114).	Psychological (CBT). Self-directed behavioural activation therapy, mindfulness, positive psychology. Comprising 12 sessions delivered over a period of 36 days.	• Findings showed a reduction in depression, hopelessness, grief, anxiety and suicide risk and a lesser effect on posttraumatic stress scores in the intervention group. • Results maintained at the 3-month post-intervention follow-up. High drop-out rate.
Elder et al.^36^ USA	To evaluate the effectiveness of online support groups for parents bereaved by cancer. To learn about bereaved parents’ grief expression in online support groups	Quantitative	Parents bereaved of a child through brain tumour cancer, (*n* = 18).	Psychoeducational/psychosocial. Delivered by trained bereavement co-facilitators. Comprising monthly on-line group meetings over a period of 20 months.	• Online support groups were considered effective; 93% (13/14) of parents reported that the group helped. About 86% of parents (12/14) said it helped to meet other parents who shared a similar experience. About 64% felt they had the opportunity to talk about my loss in a safe environment. • Newly bereaved parents focussed on pain, sadness and anger, while long-bereaved parents discussed helpful support services and changes in grief over time. Parents used online groups for a variety of reasons, including convenience, anonymity and lack of local support
Godzik et al.^37^ USA	To assess the feasibility and preliminary efficacy of utilising an online Cognitive Behavioural Therapy for Insomnia (CBT-I) programme	Quantitative RCT (online CBT-I programme versus online Psycho-educational modules on insomnia and grief.)	Bereaved adults aged 55 and older, (*n* = 30).	Psychological (CBT). Self-directed, comprising 6 core CBT-I modules, time-released over a 6-week period and includes interactive content, reading materials and sleep diaries.	• Study was feasible. About 87% retention rate, and 100% completion rate of the intervention modules. • There were no treatment effects by time difference shown and no significant differences in study outcomes were found between the CBT-I and control groups, as both demonstrated similar improvements in insomnia (*p* = 0.30).
Gold et al.^38^ USA	Study goals were: (a) to prepare for a future randomised controlled trial (RCT) on outcomes of recently perinatally bereaved moms using online support; (b) to ensure method feasibility; and (c) to gain feedback about specific components of the intervention to make it user-friendly, acceptable and culturally sensitive	Mixed methods	Perinatally bereaved mothers, (*n* = 30).	Psychoeducational/psychosocial. Self-directed with weekly links to two articles about coping with perinatal loss over a period of 6 weeks. Text message reminders were sent if participant was absent from site for more than 4 days.	• Going online helped women feel less isolated and alone and more supported, even many years after their own loss. • Participants liked having sites available in the middle of the night and that online groups could take some of the burden off of family members and friends. • Participants described the groups as safe and non-judgmental and less overwhelming than attending a face-to-face support group. • Women reported preference for anonymous groups and found it empowering to support others, although many noted an emotional cost to reading postings.
Gold et al.^39^ USA	To test feasibility of a brief pilot intervention for women of colour with perinatal loss and understand participants’ experience in an online community.	Mixed methods	Women of colour who experienced peri-natal loss, (*n* = 22).	Psychoeducational/psychosocial. Self-directed. Participants were invited to sign on and read postings at least three times weekly for 6 weeks on a specific online community for pregnancy and infant loss site.	• Feasible to recruit, retain, and track participation in an online support group for perinatally-bereaved mothers of colour. • Non-significant improvements were found in all four mental health domains (depression, posttraumatic stress disorder, moderate-severe generalised anxiety and perinatal grief). • At times, posts could increase the intensity of their loss emotions. • Grieving parents often found that friends and family are uncomfortable talking about their deceased baby and support dissipates quickly post loss. Online communities have the potential to fill this gap.
Holmgren et al.^40^ Denmark	To examine how individuals with dependent children, who had experienced death of a spouse, used an online peer support group following bereavement.	Mixed methods	Widowed parents with dependent children (*n* = 87).	Psychosocial. Self-directed, online bereavement support group (asynchronous discussion board) administered by two facilitators. Participants commented on discussion board engagement/advantages/ disadvantages.	• The use of an online peer support group in grief was experienced as highly beneficial for most, helping them to feel less alone. • 83% reported online peer support as immensely helpful. • Some described the discussion board as a ‘lifebuoy’. • One participant noted that while the support was helpful early on, later it was unhelpful and could bring back painful memories.
Kaiser et al.^41^ Germany	To evaluate a web-based cognitive behavioural intervention with asynchronous therapist support, consisting of structured writing tasks adapted specifically for prolonged grief after cancer bereavement.	Quantitative RCT (writing intervention v waitlist control)	Individuals bereaved by cancer, (*n* = 87).	Psychological (CBT). Psychologist or mental health specialist facilitated. Self-directed online grief therapy over 5 weeks with 10 structured writing tasks. Individual feedback from a therapist on writing task within 24 h	• The intervention resulted in a statistically significant reduction in prolonged grief symptoms. • High retention (93%) • Small to moderate effects on depression, anxiety, posttraumatic stress, posttraumatic growth and mental health • No impact on somatisation, sleep quality or physical health. • Those with lower social support and less posttraumatic growth experienced more prolonged grief disorder symptom change.
Kaiser et al.^42^ Germany	To examine predictors of treatment response in a secondary analysis of data from a randomised controlled trial on an internet-based intervention for prolonged grief after cancer bereavement.	Quantitative: Secondary analysis of RCT data	Individuals with prolonged grief disorder after cancer bereavement, (*n* = 70)	Psychological (CBT). Psychologist or mental health specialist facilitated. Self-directed online grief therapy over 5 weeks with 10 structured writing tasks. Individual feedback from a therapist on writing task within 24 h	• Pre-treatment symptom severity was a significant predictor of a change in grief symptoms post-intervention.
Knowles^43^ USA/Germany	To examine the feasibility and acceptability of an online interactive virtual reality (VR) support group, and assess the preliminary efficacy of the VR support group for improving psychosocial outcomes and sleep quality compared to an active control grief education website.	Quantitative Non-randomised open trial	Widow(ers) or romantic partners who have been bereaved, (*n* = 30).	Psychoeducational/psychosocial. Clinical psychologists or mental health specialists facilitated online support-support groups who met for 1 h twice per week on non-consecutive days for a total of 8 weeks (16 sessions).	• Both the VR intervention and grief education interventions were feasible and acceptable. • Both groups showed significant improvements in grief severity, grief cognitions, yearning, loneliness, perceived stress and global sleep quality across study time points. • Only widow(er)s in the Virtual Reality (VR) support group showed a significant improvement in depression across time, *p* ⩽ 0.05.
Krysinska et al.^44^ Australia	To evaluate participant and facilitator’s experiences and the perceived helpfulness of the Let’s Talk Suicide programme using qualitative interviews	Qualitative	Bereaved children, adolescents, parents, facilitators, three age groups: 7–9, 10–13 and 14–17 years old (*n* = 14).	Psychological (CBT, ACT, Narrative therapy)/ psychoeducational. Psychologist facilitated. The programme offered a 2-week online programme for children and their families. Three 1.5 h live, online group sessions and one pre-recorded parent orientation session and one live, online mid-week check-in for parents.	• The programme supported bereaved children in their grief after suicide, helped to normalise their experiences, offered support from peers and professionals and enhanced their language and skills to express themselves and deal with their emotions. • Parents felt supported in their own grief and appreciated in their role of supporting their bereaved children. • Facilitators reported that participants in the oldest age group (14–17 years) were the most difficult to engage and the most likely to drop out.
Lehmann et al.^45^ Norway	To explore the experiences of fathers whose children died unexpectedly and who were part of an online course of therapeutic writing in Norway.	Qualitative	Bereaved fathers, (*n* = 14).	Psychoeducational. Psychologist facilitated a 6-week therapeutic writing course with 3-h weekly encounters and a 30-min session of individual feedback about a chosen text. Participants were also offered two free emotional ‘first aid’ sessions with a psychologist to process the difficult emotions that would arise in the course.	• Writing became a helpful tool for meaning-making for many. • Affected processing, peer support and a dialogical platform to continue a bond with their children. • Emotional awareness, writing skills and communication skills were deepened and developed during the context of courses. • Appeared to be transformative, for those who completed the course.
Lenferink et al.^46^ Netherlands	To evaluate the efficacy of a comprehensive internet-based CBT intervention for adults bereaved due to traffic accidents experiencing elevated prolonged grief, posttraumatic stress or depression symptom levels	Quantitative RCT	Adults bereaved due to traffic accidents. (*n* = 40)	Psychological (CBT) Self-directed, comprising 8 sessions within a 12-week time frame with twice-weekly email messages from therapists. Sessions were divided into three grief tasks, (1) facing the loss and the pain that comes with it; (2) keeping faith in yourself, others, life and the future and (3) doing helpful things.	• Internet-based CBT led to significant reductions in prolonged grief, post-traumatic stress and depression symptoms at post-treatment and 8-week follow-up relative to the control. • Relatively high drop-out rate in the online CBT condition compared with the control (42% v 19%).
Lockton et al.^47^ Australia	To seek grandparents’ views in determining the usefulness of an internet-based resource for bereaved grandparents.	Quantitative: Survey	Bereaved grandparents, (*n* = 182).	Co-design. Development of an online resource in two stages. Stage 1 determined the suitability and features of an online resource through a 70-item study-specific survey. Stage 2 sought feedback on the proposed design derived from Stage 1 through a 24-question survey.	• Internet based resources for grandparents were considered useful. • Most grandparents indicated that a website with tailored resources and support would address their desire for information and provide a way to connect with other bereaved grandparents.
Pandya^48^ India	To examine the impact of an internet-based meditation programme (IMP) on socio-cultural adaptation, coping and quality of life of South Asian older widows emigrating in later life to live with their adult children in the USA.	Quantitative RCT (intervention vs an online informative game programme)	Older immigrant widows in the United States, (*n* = 172).	Psychological (meditation). Delivered by meditation experts and counsellors, arranged over a 50-week synchronous format (incorporating self-practice).	• The internet-based meditation programme (IMP) group exhibited greater socio-cultural adaptation (BSAS), adaptive coping (COPE-adaptive), enhanced quality of life and health-related quality of life (OPQOL) and decreased maladaptive coping (Brief COPE). • IMP is a meaningfully engaging intervention for late-life immigrant South Asian older widows, enabling adjustment in the new social and family contexts.
Park et al.^49^ South Korea	To explore the effects of online group art therapy on depression, grief and quality of life in Korean adults who have been bereaved.	Quantitative RCT (intervention vs no intervention control)	Korean adults who had experienced the death of an immediate family member (*n* = 36).	Psychotherapeutic/psychosocial. Online art therapy group delivered by psychologists or mental health specialists over a period of 8-weeks.	• Improvements in changes in depression, grief and quality of life from pre- to post- intervention. • Differences in depression, grief and quality of life between the art therapy and the control indicated improvements in depression and grief for the art therapy group but were not statistically significant. • Older participants unfamiliar with digital devices withdrew and expressed themselves less during the intervention.
Perluxo et al.^50^ Portugal	To explore the potential functions, perceptions and relationships between Facebook use and maternal grief following the death of a child.	Qualitative	Women who had lost their child due to accidents or prolonged illness, (*n* = 11).	Psychosocial. Self-directed access and use of Facebook during bereavement.	• Participants used Facebook to receive support, express their feelings, relate and identify with other bereaved mothers, access their child’s information and as a space to both honour and remember their child(ren).
Reitsma et al.^51^ Netherlands	To examine the short-term effectiveness of a self-guided online grief-specific cognitive behavioural therapy (CBT) in reducing early persistent complex bereavement disorder (PCBD), posttraumatic stress disorder (PTSD) and depression symptoms.	Quantitative RCT (intervention vs waitlist control)	Adults bereaved during COVID (*n* = 73).	Psychological (CBT) Self-directed grief-specific programme comprising eight weekly sessions to be completed within 12 weeks including exposure and cognitive restructuring.	• Online CBT led to significantly lower symptom-levels of persistent complex bereavement disorder, posttraumatic stress disorder and depression compared to waitlist controls. 40.6% did not complete the CBT intervention.
Smith et al.^52^ UK	To explore the impact of using online web forums for young people in the bereavement stages, following the death of a parent.	Qualitative	Young people aged between 10 and 19 years who have experienced the loss of a parent, (*n* = 10).	Psychosocial. Self-directed use of online grief support forums.	• Web-based grief support forums create an environment where young people may process the death of a parent, use their lived experience to support other bereaved young people and the space was observed to positively impact upon the young person.
Stein et al.^53^ USA	To examine the narrative accounts of 20 young adults who experienced the death of a close friend.	Qualitative	Young adults who had experienced the death of a close friend, (*n* = 20).	Psychosocial. Self-directed use of online forums of remembrance.	• Young adults’ activities both online and offline were intended to acknowledge the loss, exchange social support, create and sustain memories of their deceased friend and facilitate continued communication with the deceased. • Participants described both costs and benefits to their real and virtual remembrance activities. • Costs associated with remembrance activities were triggering overwhelming negative emotion and reminders of the reality of death.
Supiano et al.^54^ USA	To develop, implement and evaluate a distance-technology delivered grief support group programme for grieving persons in underserved rural/frontier communities in Utah.	Quantitative	Bereaved adults residing in a rural/ frontier Utah community or area (*n* = 28).	Psychoeducational/psychosocial. Social workers delivered digitally-facilitated, self-care techniques (mindfulness meditation) grief support groups over an 8-week programme.	• There was a statistically significant decrease in complicated grief and grief severity over the course of the programme. • Participants were highly satisfied with the technology and with the access to hospice service. • Several participants indicated that they felt the group and group facilitator offered ‘personal attention’ and ‘decreased social isolation’. • All but one reported that distance delivery felt the same as in-person delivery.
Sveen et al.^55^ Sweden	To examine the feasibility and preliminary efficacy of iCBT for insomnia and distress in bereaved parents 1–5 years after the loss of a child to cancer.	Quantitative RCT Intervention v active control – psychoeducation booklet)	Parents of children who died due to cancer (*n* = 21).	Psychological (CBT). Delivered by psychologists or mental health specialists. Consisted of a 9-week programme where participants completed one module per week on sleep health.	• 7 out of parents in the intervention arm who completed a telephone interview reported positive effects of the treatment. One person was dissatisfied and had not noted any improvements in sleep. • I-CBT group improved significantly from pre-to post-treatment and had a significantly larger reduction of insomnia over time. • Positive effects on prolonged grief, depression, anxiety, posttraumatic stress and grief rumination, though these results were not statistically significant.
Swartwood et al.^56^ USA	To describe peer helping behaviours occurring within internet grief forums with the goal of gaining clearer understanding of how individuals provide bereavement support online.	Mixed methods	Bereaved people accessing three internet bereavement support forums (*n* = 564 online responses).	Psychosocial. Self-directed use of bereavement support forums.	• A majority of responses contained self-disclosure. Themes in the messages shared by bereaved people suggested that online grief forums provided more than ‘one-way’ support; messages themes also included exchanging hope for the future by sharing one’s own story, validating the grief experience, providing resources and exchanging psychosocial support.
Treml et al.^57^ Germany	To examine the efficacy of an Internet-based cognitive-behavioural grief therapy (ICBGT) specifically for people bereaved by suicide.	Quantitative RCT Intervention versus waitlist control	Adults bereaved by suicide, (*n* = 58).	Psychological (CBT). Delivered by psychologists or mental health specialists. 5-week intervention with 10 writing assignments focussed on self-confrontation, cognitive restructuring and social sharing.	• Prolonged Grief Disorder symptoms decreased significantly in the treatment group compared to the waiting list group, also with reduction in grief scores. This remained stable over time, evident still at 12-month follow up. • Internet-based cognitive-behavioural grief therapy is an appropriate treatment for people with prolonged grief disorder symptoms after bereavement due to suicide.
Tur et al.^58^ Spain	To examine the effect and feasibility of an iCBT (GROw programme) for adults with Prolonged Grief Disorder.	Quantitative: Single Case multiple-baseline design	Adults with prolonged grief disorder, (*n* = 6).	Psychological (CBT). Delivered by experienced clinician. Eight modules lasting 60 min each over a period of 8–10 weeks plus a weekly support call lasting approximately 10–15 min	• Most participants (75%) showed a clinically significant change (recovered) in depression, and 50% obtained a clinically significant improvement (recovered) in symptoms of loss and typical beliefs in complicated grief. • Participants reported high usability and satisfaction with the treatment content and format
van Velsen et al.^59^ Netherlands	To share the development of an online service for coping with spousal loss (LEAVES); and to inform other developers and implementation specialists of lessons learned, while showcasing the possibilities of an eHealth tool directed at older mourners to the healthcare community.	Mixed-method development study	Older bereaved adults (*n* = 46), Stakeholders (*n* = 22).	Psychoeducational. An online self-help service that delivers the LIVIA spousal bereavement intervention, integrating an embodied conversational agent (ECA) and an initial risk assessment.	• LEAVES digital self-help service contributes to the state of the art in online grief support. • Participants viewed the main advantages of LEAVES in the possibilities of obtaining a wider range of information about the grief process and how to adapt to a world without the deceased, a lower threshold to start, the anonymity and its convenience (i.e. the independence of time and place). Low digital literacy was mentioned as an inhibiting factor to adopting LEAVES.
Wagner et al.^60^ Germany	To evaluate the treatment effects of an internet-based writing intervention for bereaved siblings aged 16–65 years	Quantitative RCT Intervention vs wait list control	People bereaved of a sibling, (*n* = 86).	Psychological (CBT). Structured writing intervention lasting 6 weeks with two 45-min assignments per week. Participants received individual therapeutic feedback for each task. Delivered by clinical psychologists.	• At post-treatment, 34% participants experienced a clinically significant improvement in grief symptoms and depression, compared to 10% in the control group. Improvements were largely maintained during one year after treatment. • Symptoms of posttraumatic stress were significantly reduced, although no significant effect was found for trauma symptoms, compared to the control group.
Wagner et al.^61^ Germany	To develop and evaluate an online group intervention for the suicide bereaved.	Quantitative RCT Intervention vs waitlist control	People bereaved through suicide, (*n* = 140).	Psychological (CBT). Delivered by psychologists or mental health specialists. A total of 12 weekly sessions, each with a duration of 90 min. Delivered in group format.	• No significant differences between the treatment and the waitlist condition were observed: pre-post intervention for depressive symptoms. • A clinically significant change could be found both for depression and grief in the intervention arm (but not significant differences were identified compared with the control). • No differences in suicidality between groups.
Weaver et al.^62^ USA	To explore the potential for offering a virtual bereaved parent support group and the perspectives of bereaved parents regarding technology acceptance and group communication dynamics.	Quantitative Pilot	Bereaved parents (*n* = 6).	Psychoeducational. 8-week online bereavement support group hosted by trained grief facilitators. Weekly group sessions lasted 1.5 h.	• All parents reported feeling benefit and satisfaction with the programme. • Respondents self-reported gaining improved communication (4/6 parents), coping (3/6 parents), peer support (3/6 parents), education (3/6 parents) and emotional expression (3/6 parents).
Wittenberg-Lyles et al.^63^ USA	To assess the potential of a Secret Facebook Group for bereaved hospice caregivers using the dual processing model of bereavement as a theoretical framework.	Mixed methods	Bereaved hospice caregivers, (*n* = 16).	Psychoeducational. Bereaved caregivers participated in a secret Facebook group following their hospice experience.	• Bereaved caregivers shared their experiences resulting in loss-orientation and shared restoration through storytelling, advising and encouragement. • Caregiver anxiety and depression scores were lower post intervention, though statistics were not reported.
Yeates et al.^64^ Australia	To co-design an online support intervention for families after sudden cardiac death (SCD) in the young.	Qualitative Co-design	Families (*n* = 6), healthcare professionals (*n* = 5) and a peer researcher (*n* = 1) with experience in Sudden Cardiac Death (SCD) in the young, (*n* = 12).	Psychosocial/psychoeducational Moderated by a clinical psychologist or genetic counsellor. Focus groups held via video-conference over a period of 4 months.	• Service providers and end user group in this codesign process were highly motivated to participate and deep insights into the experiences of families following sudden cardiac death shaped the priorities and specific interventions developed. • The process resulted in an intervention that families believe will be of assistance in the most tragic of circumstances and one that will equip healthcare professionals with practical and helpful tools so they may provide better support.
Yu et al.^65^ China	To evaluate the ‘Be Together Programme’ (BTP), a new psychosocial grief intervention approach developed for Wuhan COVID-19 bereaved people.	Quantitative	Family members bereaved due Covid-19, (*n* = 45).	Psychological/psychosocial/psychoeducational. Delivered by social workers and mental health specialists. Participants invited to attend a group ‘Supermarket Mode’, broad ‘shopping list’ of choices incorporating loss-oriented and restoration-oriented themes. Participants also received approximately 15 h of online one-to-one support from social workers.	• Statistically significant improvements in complicated grief did not occur between Time 1 and Time 2 (approximately 5 months) but were found from Tine 2 to Time 3 (approximately 12 months post T1). • Impacts were greater for those with prolonged grief disorder.

### Evidence quality

Of four systematic reviews, one was high quality,^12^ two were moderate quality^13,14^ and one was low quality.^15^ Most primary research studies were judged as high (*n* = 20) or moderate quality (*n* = 10), with few low-quality studies (*n* = 5; Supplemental Material File 1). We did not exclude studies based on their quality; instead we included all studies to allow for a comprehensive understanding of the evidence. We refer to quality when reporting findings from the systematic review papers in the relevant sections below; and report the quality score allocated to each individual study in our Supplementary material file.

### Findings from systematic reviews

Four systematic reviews examining online bereavement support were included (Table 3).^12–15^ These were published between 2019 and 2021, with all included studies conducted before the COVID-19 pandemic. One review focussed on bereavement following suicide^14^ and three were focussed on support for adults irrespective of the type of bereavement.^12,13,15^ Two reviews included quantitative, qualitative and mixed-methods study designs,^13,14^ one evaluated randomised controlled trials (RCTs) and feasibility studies,^12^ while one included RCTs only.^15^

Interventions based on cognitive behavioural therapy (CBT) were the focus of two reviews.^12,15^ One review, evaluated as low-quality, included seven RCTs focussed on web-based CBT;^15^ their meta-analysis revealed moderate to large effects relating to improvements in symptoms of grief and posttraumatic stress disorder for those receiving online CBT compared to those in a control group. A statistically significant improvement in depression was noted, though the effect was small. A higher number of treatment sessions was linked with greater impact. The other, a high-quality review, included nine studies,^12^ seven of which were the same as those evaluated by first review described.^15^ All but one were CBT-based interventions. The non-CBT intervention was a psychoeducational online resource based on social cognitive theory. Both reviews found that online bereavement support resulted in statistically significant improvements in symptoms of grief, depression and posttraumatic stress compared to a control group. User satisfaction was reported in over half of the online CBT-informed interventions analysed in the high-quality review,^12^ and was typically rated as moderate to high.

The two reviews examining online support groups and forums included a variety of study designs – quantitative, qualitative and mixed method.^13,14^ Both reviews were evaluated as moderate quality. The integrative review,^14^ concluded that online resources offer around-the-clock support for people who have been bereaved by suicide and are helpful to those who are seeking and sharing support and/or information, memorialising their loved ones and meaning-making. Negative effects were rare. The other, a systematic review,^13^ examined online support for bereaved adults, irrespective of the type of loss. It identified positive impacts of online peer support including being part of an understanding community of people who share similar experiences, access to emotional support, information sharing, remembrance, reconstruction of identity and acknowledgement of the how grief changes over time.

Taken together, evidence from four systematic reviews indicate the value of online bereavement support interventions. These include positive impacts on bereavement outcomes, 24-hour access to bereavement support and improved access to support for people in remote areas and those that may not otherwise receive support. However, the reviews also conclude that evidence, in particular quantitative evidence was not high quality, and that further rigorous research is needed to strengthen the evidence base.

### Findings from primary studies

Thirty-five papers, not previously identified for inclusion in the four systematic reviews, were synthesised. These were published between 2011 and 2023, with a noticeable increase in the number published from 2021 onwards. Studies were conducted by academic teams based in the USA (*n* = 11), Europe (*n* = 16), Australia (*n* = 3) and one each in China, India and South Korea. A further two papers involved teams from more than one country. Of the 16 European papers, five were from Germany, three from the Netherlands and one each from Denmark, Italy, Norway, Portugal, Spain, Sweden, Switzerland and the UK (Table 4).

Study designs were quantitative (*n* = 20), qualitative (*n* = 8) and mixed method (*n* = 7). Most focussed on evaluation (*n* = 22), while a sub-set were specifically development studies (*n* = 5), or feasibility/pilot studies (*n* = 8). In terms of study populations, nearly half did not specify any type or cause of bereavement (*n* = 16). Four studies were focussed on support for people bereaved following suicide; six involved people bereaved due to cancer or a long-term condition; three studies examined support following bereavement during the COVID-19 pandemic, three examined peri-natal loss and three focussed on bereavement support following sudden or unexpected death (e.g. road traffic accident or sudden cardiac arrest in a young person). See Table 4.

Over 40% of studies (*n* = 15) did not specify the relationship between the deceased and bereaved person, however others examined specific relationships such as parent of the deceased (*n* = 8, including one study on bereaved fathers and one on bereaved mothers), spouse or partner (*n* = 4), any family member (*n* = 4, including one study focussed on children who had lost a close family member), grandparent of the deceased (*n* = 1), child or young adult who had been bereaved of a parent (*n* = 1), close friend (*n* = 1) or sibling (*n* = 1).

### Intervention type and format

Interventions were broadly categorised as psychological, psychoeducational, psychosocial or unspecified/in development, though there were many overlaps. CBT was the most common psychological approach used as a basis for online interventions (*n* = 13).^31,35,37,41,42,44,46,51,55,57,58,60,61^ Theories of grief such as task-based approaches to mourning, and the Dual Process Model formed the basis for a small number of interventions.^31,63,65^ Psychoeducational interventions aimed to share information on grief and coping skills, and normalising grief experiences.^32,62,63^ Psychosocial interventions included online forums and interventions that aimed to promote peer support and connection with others.^33,40,50,52,53,56,65^ Many interventions included both psychosocial and psychoeducational components.^32,34,36,38,39,43,54,64^ A small number of development studies did not specify an intervention type from the outset.

The format of interventions could be broadly classified as self-directed (*n* = 14), individual (*n* = 11) or group-based (*n* = 10; Table 4). Self-directed interventions included engagement with online forums and self-guided access to online CBT with little or no feedback. Individual interventions included live-chat conversations, CBT with individualised feedback and one-to-one support delivered online. Group interventions were typically bereavement support group sessions delivered online and facilitated by a trained volunteer or professional.

### Effectiveness of online bereavement support

Across all studies using quantitative measures, there was good evidence that online bereavement interventions result in statistically significant improvements in a range of outcomes (Table 4). These include grief symptoms or severity,^31,35,37,43,44,49,51,54,58,60,61^ depression,^35,37,41,43,46,49,51,58,60,61^ anxiety^35,41,55^ stress or posttraumatic stress,^35,41,43,46,51,60^ insomnia,^37,43,55^ coping^31,48^ and mental health or quality of life.^37,41,48^ Of these, five studies reported improvements in grief outcomes versus a control condition,^35,41,46,57,61^ providing strong evidence of effect compared with those studies comparing outcomes based on pre-post designs or within-group changes over time. Other studies reported improvements in outcomes that were not statistically significant, often due to small sample sizes. For example, four studies reported improvements in grief outcomes that were not statistically significant.^39,49,55,65^ No studies reported worsening of outcomes following online bereavement support. Several studies alluded to cost-savings, low-costs or cost-effectiveness of online interventions, but did not specify the types of costs and cost-savings.

### Acceptability of online bereavement support

Acceptability relates to the individual’s perceived satisfaction with an intervention in relation to addressing a problem. Overall, online interventions for bereavement support were judged to be acceptable. Several studies described overall benefits and positive impacts of online interventions indicating appropriateness and acceptability.^31–34,36,38–41,43–46,48–55,59^ Such benefits included: access to a supportive community and reduced isolation^32,33,36,38,39,40,43,44,47,50,52–54,62^; opportunities to express and process one’s feelings around grief;^36,38,40,44,50,52,56,62^ normalising of feelings and experiences in response to loss;^40,44,56^ information on coping strategies;^36,40,44,45,48,59,62^ meaning-making;^33,44,45^ and opportunities to remember the person who died.^45,50,53^

People who had been bereaved described online forums as a safe space in which they could disclose personal information.^33,36,49,52^ Some experienced online support as less overwhelming than attending an in-person face-to-face support group.^38^ People bereaved due to suicide found online ‘live chat’ a safe space to disclose non-socially desirable details relating to their loss and to make sense of suicide through the reconstruction of events and the motivations of the person who died.^33^

### Effectiveness and acceptability by intervention format

For all types of online support formats, assessed via quantitative, qualitative and mixed method designs, positive impacts were identified. Group-based interventions were perceived as beneficial,^32,43,44,62^ helped people cope and feel less alone,^38,43^ supported communication^62^ and provided opportunities for longer standing group members to advise and support new members.^44^ Individual interventions helped reduce grief symptoms^61^ and prolonged grief disorder;^41,57,65^ promoted adapted coping,^48^ and supported emotional processing and meaning-making.^33,45^ Self-guided interventions, which included self-guided formal interventions such as CBT, as well as unguided access to intervention forums and chat groups were also linked with positive impacts and general benefits. These included reduced grief symptoms,^35,46,51,56^ improved coping,^31,52^ access to a supportive community,^47,52^ opportunities to remember the person who died^50,53^ and general perceptions of benefit.^40^

### Impact on access to bereavement support

Online bereavement support was found to facilitate access to support, especially for people who lived in remote or rural areas, or areas where local support was unavailable.^32,34,54^ Many online interventions are available 24/7, enabling support at any time.^36,39^ Online forums and communities were found to provide early access to bereavement support before people have managed to arrange access to more formal or structured support.^34,50^ Online interventions were also viewed as convenient and flexible, with individuals accessing the support they needed, when they needed it.^32,36,61^ For some bereaved individuals, online interventions were valued as they enabled anonymous engagement.^33,36,38,43,57^ There was evidence that online support could be beneficial to people of all ages including older individuals.^31,37,43,48^ One study successfully recruited older adults for a feasibility study examining online CBT versus a control condition for insomnia, mood and quality of life.^37^ Mean age of participants was 65 years (range = 55–84 years), though most were white educated women, based in the US with access to a computer and already using social media.^37^ Another found evidence for the feasibility and effectiveness of an online virtual reality support group for widow and widowers who had a mean age of 67 years, though the sample consisted of mostly white female participants, based in the US.^43^ One study examining the impact of an internet-based meditation programme on coping and quality of life amongst late-life immigrant older widows concluded that an internet-based meditation programme was effective.^48^ However, eligibility criteria included self-reported comfort using digital technology and smartphones at the time at the time of recruitment (in 2017).

### Negative impacts and unintended consequences

Few risks or negative impacts were identified. Those that were reported were mainly unintended consequences linked with unmoderated online forums. Such impacts included possible increases in grief intensity on reading posts about other people’s experiences of loss online^39^ or upset due to inappropriate or insensitive online comments.^50^ A lack of personal contact associated with unmoderated forums such as Facebook was also perceived as negative in comparison with other forms of support.^50^ Recognising these issues, one study examining web forums for young people who had been parentally bereaved recommended guiding young people to information regarding online safely when using bereavement support web forums.^52^

### Intervention feasibility and retention rates

We examined retention rates as an indicator of intervention feasibility. Retention rates and/or dropout rates were available for about half of all studies (*n* = 16). Overall, retention rates were high – most studies (11 of 16) reported retention rates greater than 70% and all but one^35^ reported retention rates over 50%. For instance, an RCT examining the impact of a therapy-assisted web-based writing intervention for prolonged grief disorder reported overall drop-out rates of only 7% (6 of 87 participants).^41^ The intervention consisted of 10 structured writing tasks that participants worked on independently in two self-scheduled 45-min sessions per week. Within 24 h of each session, participants received feedback from a trained psychologist. In a secondary data analysis linked with this study,^42^ the authors reported that therapeutic alliance (favourable relationship between therapist and client) predicted improvement in symptoms of prolonged grief disorder and suggested this is also likely to have had a positive impact on retention.

Only one study highlighted unusually high levels of drop-out.^35^ This was an efficacy RCT of an unguided web-based grief intervention for adults who had been bereaved during the COVID-19 pandemic. Of 882 allocated to the intervention, only 45 completed it, and of those, more than half were lost to follow-up. The authors suggested this may have been due to the online self-guided nature of the intervention, a preference for face-to-face interventions or due to the length of the intervention (12 modules delivered over 36 days, in video or text format). There was also a delay between participant consent and access to the online support, during which many participants withdrew.

Facilitation varied across interventions from no facilitation, to asynchronous feedback;^41,42,46,57^ real time volunteer or co-facilitator facilitation;^33,36^ or real-time online support from a therapist or mental health specialist.^32,43,44^ Retention was lowest for unguided interventions^35^ and highest for interventions involving greater levels of facilitation.^41,42^

### Barriers and facilitators of online bereavement support

Factors linked with the implementation of online bereavement support included technical issues and mode of facilitation or support. Technical barriers included challenges typing information or feedback, difficulties controlling sound and hearing audio-visual material, or difficulties logging into a website.^37,38^ Irrelevant or repetitive material was also a barrier to engagement.^37^ Sending feedback or reminders to individuals to keep going with the intervention could promote engagement.^34,41^ For unguided interventions, instructions need to be simple and clear, and need to take account of different cultural norms.^48^ Information also needs to be focussed on topics relevant to the individual and easy to access.^37^ For interventions that involved therapist feedback or input, the quality of therapist engagement or ‘therapeutic alliance’ was considered important, and needs to be fostered to support intervention acceptability and effectiveness.^42^ While IT literacy and language were occasionally mentioned as a possible barrier,^34^ no studies explored this in any detail.

## Discussion

Our review of evidence from 4 reviews and 35 individual studies indicates that online bereavement support is an acceptable form of bereavement support for many, and can lead to improvements in grief intensity, depression and stress-related outcomes. Retention rates for formal interventions were typically over 70%, with most bereaved participants completing the intervention once they started. Few risks of online bereavement support were identified. Where reported, such risks tended to relate to informal or non-moderated forms of support such as chat groups where risks included increased grief intensity on exposure to the loss of others; insensitive online comments; or spending too much time online reflecting on loss. However, in general negative impacts were outweighed by positive impacts such as access to a supportive community, space to reflect and share experiences safely, access to information on coping and opportunities to remember the person who had died.

Positive impacts were identified for all formats, from self-guided to individual-facilitated and group interventions, with many of the impacts described also closely resembling those identified in a recent review of predominantly in-person bereavement interventions.^66^ This suggests that there is potential value in diverse forms of online bereavement support with different formats appealing to different individuals depending on their preferences and needs. For those seeking a supportive community, online group-based interventions or online forums may serve their needs.^13,14^ For those struggling with specific grief symptoms or prolonged grief disorder, online interventions offering individualised therapist-facilitated support may be most suitable. For instance, online CBT based interventions delivered by a therapist, can be of particular benefit to those experiencing prolonged grief disorder and related problematic symptoms.^12,15^ National bereavement support guidance needs to reflect the potential value of online interventions to increase access to bereavement support for people with varying levels of needed, who might not otherwise be able to benefit.

While the findings from this review are generalisable only to those that have accessed online bereavement support, our results suggest that once accessed, online interventions are generally perceived as acceptable across a range of populations. This includes young people,^52^ older adults,^31,37,47,48,59^ bereaved parents,^34,36,38,39,45,50,55,62^ people bereaved due to chronic conditions,^36,41,54,55,63^ as well as those bereaved due to suicide and traumatic circumstances.^33,44,57,61,64^ Overall retention was typically above 70%, and in line with retention rates for online interventions targeting mental health problems.^67^ While most studies described good levels of acceptability overall, there was some evidence that particular sub-groups found it less easy to engage in some online formats. For instance, a study of online art therapy reported that bereaved older adults were less likely to express themselves and were more likely to withdraw.^49^ Barriers to uptake of online interventions for older adults have been well documented, but can be addressed through active engagement of this group in intervention design, leading to increased self-efficacy in use of technology and knowledge in relation to security concerns.^68^

Our findings align with results from reviews of online interventions for mental health and wellbeing for other populations. A meta-review of 304 studies found that digital health interventions offer promise in treating a range of problems including substance use-, anxiety-, mood- and trauma-related disorders.^69^ Specifically, they offer greater comfort and flexibility than in-person visits, while retaining usefulness.^69^ Similarly, systematic review evidence from 43 studies examining online interventions for anxiety and depression reported that online interventions are generally effective in reducing symptoms, with impacts being enhanced where there is therapist input.^70^ There is also evidence that unguided interventions can be effective, with a review of 32 studies focussed on self-guided online interventions aimed at preventing anxiety and depression, revealing that self-guided interventions have a small but meaningful impact on anxiety and depression.^71^ The same review found that unguided interventions may lead to a reduced risk of receiving a diagnosis of depression for certain populations, though evidence regarding preventing anxiety was inconclusive. Despite a need for more rigorous evidence, there is a growing consensus from this review and the wider literature that online interventions aiming to improve mental health and wellbeing outcomes, are acceptable and effective.

## Strengths and limitations

This rapid integrative review provides a comprehensive and timely synthesis of the evidence relating to online bereavement support. A large team of co-authors facilitated rapid data extraction and quality appraisal of eligible papers. Our inclusion criteria were intentionally broad to allow exploration of a range of online interventions, including formal and informal bereavement support. However, the heterogeneity of study populations, intervention types and outcomes across studies meant that a sub-analysis of impact according to different intervention formats was not possible.

## Implications for intervention development and practice

Our findings suggest that online interventions could play a role in improving access to bereavement support for different levels of need.^72–74^ This could be through self-guided information and support; online interventions facilitated by volunteers, healthcare professionals and community groups; as well as online interventions delivered by clinical psychologists or mental health specialists to support people experiencing problematic grief or prolonged grief disorder.

Online delivery of bereavement support enables the provision of bereavement support to those who might not otherwise have access, or choose to access, such support. This includes those in remote and rural areas,^32,54^ those who prefer anonymity when seeking support,^14,33,36,39,43,57^ and those who need convenient and flexible access, or support outside of usual working hours.^36,56^ By signposting to, and offering online bereavement support, organisations which provide bereavement support can increase their reach and impact.

The degree of moderation or facilitation needs careful consideration when developing online bereavement support. In general, retention is lowest for unguided interventions^35^ and highest for those that involve some level of facilitation.^41,42^ Personalised feedback, information regarding intervention content or effectiveness, reminders, rewards and feedback about progress was linked to participant retention in the studies evaluated here, and are known to promote engagement with web-based mental health programs in general.^75^ To minimise any potential negative impacts from engagement with online forums, use of a forum moderator needs to be considered. Moderators can help create a cohesive culture of empathy, sensitivity and care in a safe community,^76^ but also need individual and organisational-level support to operate effectively and maintain their own wellbeing.^77^

### Directions for future research

Very few studies reported the recruitment or response rate, that is the number of eligible participants versus the number who consented to take part. This is often impossible to determine for studies conducted in applied settings as researchers often do not know the total number of people who had sight of a study invitation or advert. However, this can result in non-response bias, whereby those who were recruited are systematically different from those not recruited, and therefore not representative of the general population.^78^ Inequalities in access to bereavement support are well-documented with minoritised ethnic groups, LGBTQ+ communities and people with lower socio-economic status more likely to experience barriers to support.^79–81^ Given the lack of data on response rates, it is not possible to gauge the extent to which diverse groups of individuals may have experienced barriers to participation. Across most studies, white females were overrepresented, reflecting the characteristics of participants in previous bereavement interventions^82^ and UK national bereavement surveys.^79,83^ Future studies need to engage more diverse communities so that findings are applicable to a wider range of individuals. No studies focussed on socio-economically deprived or LGBTQ+ communities were identified.

Our review reveals that the evidence for online bereavement support is rapidly emerging, but several gaps can be identified. First, there is a need for more studies examining the effectiveness of online bereavement support compared to a control group. Pre- and post-studies provide weaker evidence as they can’t control for the possibility that improvements may occur with the passing of time, as opposed to engagement with the intervention. Second, most research participants tend to be white females – there is a need to develop and evaluate online interventions with diverse populations, in particular those groups that are typically underserved.^84,85^ Research that engages minoritised ethnic groups, LGBTQ+ people and individuals from different cultural and faith backgrounds is urgently needed, and individuals that are to be served by the intervention, need to be involved in its design from the outset. Third, research is needed to explore the role of moderators and facilitators of online bereavement support interventions, and their relationship with key outcomes such as coping and quality of life for people seeking support.^86^ Fourth, research that involves children and young people in the development of engaging online support needs to be conducted. Fifth, despite the assumption that online interventions are more cost-effective than in-person interventions, we did not identify any health-economic data to support this. Research examining the costs and cost-savings associated with different forms of online bereavement support is urgently needed and needs to take account of indirect costs such as impact on physical health, ability to work and addiction. Finally, future research might usefully adopt implementation science approaches to examine potential barriers and facilitators relating to accessing online bereavement support, and consider steps that can be taken to maximise inclusivity.^87^

## Conclusion

Online interventions can widen access to acceptable, effective bereavement support and improve outcomes for people who have been bereaved. Negative or unintended consequences of online bereavement support were mainly linked with unmoderated forums, but these were infrequently reported. Future research examining the role of online intervention facilitation and moderation is recommended, as are studies involving more diverse populations and assessing cost-effectiveness. National policies and clinical guidelines relating to bereavement support need to be updated to take account of the wide variety of online bereavement support formats that are now available.

## Supplemental Material

sj-docx-1-pmj-10.1177_02692163241285101 – Supplemental material for A rapid review of the evidence for online interventions for bereavement support Supplemental material, sj-docx-1-pmj-10.1177_02692163241285101 for A rapid review of the evidence for online interventions for bereavement support by Anne Finucane, Anne Canny, Ally Pax Arcari Mair, Emily Harrop, Lucy E Selman, Brooke Swash, Donna Wakefield and David Gillanders in Palliative Medicine
