# Supplementary material for: A rapid review of the evidence for online interventions for bereavement support
Source: Palliat Med. 2024 Oct 15;39(1):31–52. doi: 10.1177/02692163241285101 (PMC11673319; doi:10.1177/02692163241285101)
Supplement: sj-docx-1-pmj-10.1177_02692163241285101 – Supplemental material for A rapid review of the evidence for online interventions for bereavement support [file sj-docx-1-pmj-10.1177_02692163241285101.docx]

**Supplementary File 1. Summary of quality appraisal for systematic reviews and primary data studies.**

| **Summary of Quality Appraisal – Systematic Reviews by AMSTAR** | | | | | |  |
| --- | --- | --- | --- | --- | --- | --- |
|  |  | Lestienne et al. 2021 | Robinson & Pond 2019 | Wagner et al. 2020 | Zuelke et al. 2021 | |
| **1** | Did the research questions and inclusion criteria for the review include the components of PICO? | yes | yes | yes | yes | |
| **2** | Did the report of the review contain an explicit statement that the review methods were established prior to the conduct of the review and did the report justify any significant deviations from the protocol? | partial | partial | partial | partial | |
| **3** | Did the review authors explain their selection of the study designs for inclusion in the review? | yes | yes | no | no | |
| **4** | Did the review authors use a comprehensive literature search strategy? | partial | partial | partial | partial | |
| **5** | Did the review authors perform study selection in duplicate? | yes | no | no | yes | |
| **6** | Did the review authors perform data extraction in duplicate? | yes | no | yes | yes | |
| **7** | Did the review authors provide a list of excluded studies and justify the exclusions? | partial | partial | partial | yes | |
| **8** | Did the review authors describe the included studies in adequate detail? | partial | partial | yes | yes | |
| **9** | Did the review authors use a satisfactory technique for assessing the risk of bias (RoB) in individual studies that were included in the review? | partial | partial | partial | partial | |
| **10** | Did the review authors report on the sources of funding for the studies included in the review? | no | no | no | yes | |
| **11** | If meta-analysis was performed did the review authors use appropriate methods for statistical combination of results? | N/A | N/A | yes | yes | |
| **12** | If meta-analysis was performed, did the review authors assess the potential impact of RoB in individual studies on the results of the meta-analysis or other evidence synthesis? | N/A | N/A | no | yes | |
| **13** | Did the review authors account for RoB in individual studies when interpreting/ discussing the results of the review? | yes | yes | yes | yes | |
| **14** | Did the review authors provide a satisfactory explanation for, and discussion of, any heterogeneity observed in the results of the review? | yes | yes | yes | yes | |
| **15** | If they performed quantitative synthesis did the review authors carry out an adequate investigation of publication bias (small study bias) and discuss its likely impact on the results of the review? | N/A | N/A | no | yes | |
| **16** | Did the review authors report any potential sources of conflict of interest, including any funding they received for conducting the review? | yes | yes | no | yes | |
|  | Overall Quality Rating | Moderate | Moderate | Low | High | |

= Critical domains of AMSTAR2 https://www.ncbi.nlm.nih.gov/pmc/articles/PMC5833365/

The link describes AMSTAR2 article and gives the following guidance for assessing overall quality: Firstly, if no meta-analysis was conducted in the review, then omit critical item 11 and 15. We agreed not to penalise partial scores.

Rating overall confidence in the results of the review as follows:

**High:** No or one non-critical weakness: the systematic review provides an accurate and comprehensive summary of the results of the available studies that address the question of interest.

**Moderate:** More than one non-critical weakness*****: the systematic review has more than one weakness but no critical flaws. It may provide an accurate summary of the results of the available studies that were included in the review.

**Low:** One critical flaw with or without non-critical weaknesses: the review has a critical flaw and may not provide an accurate and comprehensive summary of the available studies that address the question of interest.

**Critically low:** More than one critical flaw with or without non-critical weaknesses: the review has more than one critical flaw and should not be relied on to provide an accurate and comprehensive summary of the available studies.

***** Multiple non-critical weaknesses may diminish confidence in the review and it may be appropriate to move the overall appraisal down from moderate to low confidence.

| **Summary of Quality Appraisal of Primary Data Studies by MMAT** | | | | | | | |
| --- | --- | --- | --- | --- | --- | --- | --- |
| **Qualitative** | **S1** | **S2** | **1.1** | **1.2** | **1.3** | **1.4** | **1.5** |
|  | Are there clear research questions? | Do the collected data allow to address the research questions? | Is the qualitative approach appropriate to answer the research question? | Are the qualitative data collection methods adequate to address the research question? | Are the findings adequately derived from the data? | Is the interpretation of results sufficiently substantiated by data? | Is there coherence between qualitative data sources, collection, analysis and interpretation? |
| Cipolletta, *et al.* (2021) | Yes | Yes | Yes | Yes | Yes | Yes | Yes |
| Dias *et al. (*2021) | No | Yes | Yes | No | Yes | No | Yes |
| Krysinska *et al*. (2023) | Yes | Yes | Yes | Yes | Yes | Yes | Yes |
| Lehmann *et al*. (2023) | Yes | Yes | Yes | Yes | Yes | Yes | Yes |
| Lockton *et al*. (2023) | Yes | Yes | Yes | No | Yes | No | Yes |
| Perluxo D., Francisco R. (2018) | Yes | Yes | Yes | Yes | Yes | Yes | Yes |
| Smith K., Langer S. (2021) | Yes | Yes | Yes | Yes | Yes | Yes | Yes |
| Stein *et al*. (2018) | Yes | Yes | Yes | Yes | Yes | Yes | Yes |
| van Velsen *et al*. (2023) | Yes | Yes | Yes | Yes | CT | Yes | No |
| Yeates *et al.* (2021) | Yes | Yes | Yes | CT | CT | CT | CT |
| **Quantitative randomised controlled trials** | **S1** | **S2** | **2.1** | **2.2** | **2.3** | **2.4** | **2.5** |
|  | Are there clear research questions? | Do the collected data allow to address the research questions? | Is randomization appropriately performed? | Are the groups comparable at baseline? | Are there complete outcome data? | Are outcome assessors blinded to the intervention provided? | Did the participants adhere to the assigned intervention? |
| Brodbeck *et al*. (2022) | Yes | Yes | Yes | Yes | Yes | CT | Yes |
| Dominguez-Rodriguez *et al*. (2023) | Yes | Yes | Yes | Yes | Yes | Yes | Yes |
| Godzik C *et al*. (2021) | Yes | Yes | Yes | Yes | Yes | CT | Yes |
| Kaiser *et al*. (2023) | Yes | Yes | Yes | Yes | Yes | No | Yes |
| Kaiser *et al*. (2022) | Yes | Yes | Yes | Yes | Yes | Yes | Yes |
| Lenferink *et al*. (2023) | Yes | Yes | Yes | Yes | No | Yes | No |
| Pandya SP. (2021) | Yes | Yes | Yes | Yes | Yes | Yes | Yes |
| Park SR., Cha Y. (2023) | Yes | Yes | Yes | No | Yes | CT | No |
| Reitsma *et al*. (2023) | Yes | Yes | Yes | Yes | No | CT | No |
| Treml *et al*. (2021) | Yes | Yes | Yes | Yes | Yes | No | Yes |
| Wagner *et a*l. (2022) (a) | Yes | Yes | Yes | Yes | No | No | No |
| Wagner *et al*. (2022) (b) | Yes | Yes | Yes | Yes | No | CT | Yes |
| **Quantitative non-randomised controlled trials** | **S1** | **S2** | **3.1** | **3.2** | **3.3** | **3.4** | **3.5** |
|  | Are there clear research questions? | Do the collected data allow to address the research questions? | Are the participants representative of the target population? | Are measurements appropriate regarding both the outcome and intervention (or exposure)? | Are there complete outcome data? | Are the confounders accounted for in the design and analysis? | During the study period, is the intervention administered (or exposure occurred) as intended? |
| Elder J., Burke L. (2015) | Yes | Yes | Yes | Yes | Yes | No | CT |
| Knowles *et al*. (2017) | Yes | Yes | Yes | Yes | Yes | No | Yes |
| Supiano *et al*. (2021) | Yes | Yes | Yes | Yes | Yes | CT | Yes |
| Yu *et al*. (2022) | Yes | Yes | Yes | Yes | No | CT | Yes |
| **Quantitative descriptive** | **S1** | **S2** | **4.1** | **4.2** | **4.3** | **4.4** | **4.5** |
|  | Are there clear research questions? | Do the collected data allow to address the research questions? | Is the sampling strategy relevant to address the research question? | Is the sample representative of the target population? | Are the measurements appropriate? | Is the risk of nonresponse bias low? | Is the statistical analysis appropriate to answer the research question? |
| Tur *et al*. (2022) | Yes | Yes | No | No | No | Yes | No |
| Weaver *et al*. (2021). | Yes | No | Yes | No | Yes | No | Yes |
| **Mixed methods** | **S1** | **S2** | **5.1** | **5.2** | **5.3** | **5.4** | **5.5** |
|  | Are there clear research questions? | Do the collected data allow to address the research questions? | Is there an adequate rationale for using a mixed methods design to address the research question? | Are the different components of the study effectively integrated to answer the research question? | Are the outputs of the integration of qualitative and quantitative components adequately interpreted? | Are divergences and inconsistencies between quantitative and qualitative results adequately addressed? | Do the different components of the study adhere to the quality criteria of each tradition of the methods involved? |
| Chang *et al*. (2016) | Yes | Yes | Yes | Yes | No | Yes | No |
| Gold *et al*. (2021) | Yes | Yes | Yes | Yes | Yes | Yes | Yes |
| Gold *et al*. (2022) | Yes | Yes | Yes | Yes | Yes | Yes | Yes |
| Holmgren, H. (2023) | Yes | Yes | Yes | Yes | Yes | Yes | CT |
| Sveen *et al*. (2021) | Yes | Yes | Yes | Yes | Yes | CT | CT |
| Swartwood *et al*. (2011) | Yes | Yes | Yes | Yes | Yes | Yes | Yes |
| Wittenberg-Lyles *et al*. (2015) | Yes | Yes | Yes | Yes | Yes | Yes | Yes |

| **Author** | **Quality** |
| --- | --- |
| Cipolletta, S., Entilli L., Bettio F., De Leo D. (2021) | ***** |
| Dominguez-Rodriguez A., Sanz-Gomez S., Ramirez LPG., *et al*. (2023) | ***** |
| Gold K., Boggs M., Kavanaugh K. (2021) | ***** |
| Gold K., Boggs E., Plegue A., Andalibi N. (2022) | ***** |
| Kaiser J., Nagl M., Hoffman R., Linde K., Kersting A. (2022) | ***** |
| Krysinska K., Currier D., Andriessen K. (2023) | ***** |
| Lehmann O., Kalstad TG., Neimeyer RA. (2023) | ***** |
| Pandya SP. (2021) | ***** |
| Perluxo D., Francisco R. (2018) | ***** |
| Smith K., Langer S. (2021) | ***** |
| Stein CH., Hartl Majcher J., Froemming MW., Greenberg SC., Benoit MF., Gonzales SM., *et a*l. (2018) | ***** |
| Swartwood R., McCarthy Veach P., Kuhn J., Kyung Lee H., Kangting J. (2011) | ***** |
| Wittenberg-Lyles E., Washington K., Parker Oliver D., Shaunfield S., Cage LA., Mooney M., Lewis A. (2015) | ***** |
| Brodbeck J., Berger T., Biesold N., Rockstroh F., Schmidt S., Znoj H. (2022) | **** |
| Godzik C., Crawford S., Ryan E. (2021) | **** |
| Holmgren H. (2023) | **** |
| Kaiser J., Treml J., Hoffmann R., Linde K., Nagl M., Kersting. (2023) | **** |
| Knowles L., Stelzer EM., Jovel KS., O'Connor MF. (2017) | **** |
| Supiano P., Koric A., Jacob E. (2021) | **** |
| Treml J., Nagl M., Linde K., Kundige C., Peterhansel C., Kersting A. (2021) | **** |
| Chang J., Sequeira A., McCord C., Garne W. (2016) | *** |
| Elder J., Burke L. (2015) | *** |
| Lenferink LIM., Eisma MC., Buiter MY., de Keijser J., Boelen PA. (2023) | *** |
| Lockton J., Oxlad M., Due C. (2023) | *** |
| Sveen JB., Jernelov S., Pohlkamp L., Kreicbergs U., Kaldo V. (2021) | *** |
| van Velsen L., Schokking L., Siderakis E., Knospe G., Brandl L., Mooser B., *et al*. (2023) | *** |
| Wagner B., Grafiadeli R., Schafer T., Hofmann L. (2022) (b) | *** |
| Yu Z., Liang J., Guo L., Jiang L., Wang J., Ke M., *et al*. (2022) | *** |
| Dias N., Boring E., Johnson L., Grossoehme DH., Murphy S., Friebert S. (2021) | *** |
| Weaver MS., Jurens A., Neumann MA., *et al.* (2021) | *** |
| Park SR., Cha Y. (2023) | ** |
| Reitsma L., Boelen PA., de Keijser J., Lenferink LIM. (2023) | ** |
| Wagner B., Hofmann L., Maass U. (2022) (a) | ** |
| Tur C., Campos D., Suso-Ribera C., Kazlauskas E., Castilla D., Zaragoza I., *et al*. (2022) | * |
| Yeates L., Gardner K., Do J., van den Heuvel L., Fleming G., Semsarian C., *et al*. (2021) | * |
